# The Increase of Soft Cheese Shelf-Life Packaged with Edible Films Based on Novel Hybrid Nanostructures

**DOI:** 10.3390/gels8090539

**Published:** 2022-08-26

**Authors:** Aris E. Giannakas, Constantinos E. Salmas, Dimitrios Moschovas, Konstantinos Zaharioudakis, Stavros Georgopoulos, Georgios Asimakopoulos, Anastasios Aktypis, Charalampos Proestos, Anastasios Karakassides, Apostolos Avgeropoulos, Nikolaos E. Zafeiropoulos, George-John Nychas

**Affiliations:** 1 Department of Food Science and Technology, University of Patras, 30100 Agrinio, Greece; 2 Department of Material Science and Engineering, University of Ioannina, 45110 Ioannina, Greece; 3 School of Food and Nutritional Sciences, Department of Food Science and Human Nutrition, Laboratory of Microbiology and Biotechnology of Foods, Agricultural University of Athens, Iera Odos 75, 11855 Athens, Greece; 4 Laboratory of Food Chemistry, Department of Chemistry, National and Kapodistrian University of Athens, 15771 Athens, Greece

**Keywords:** active packaging, soft cheese preservation, sodium alginate, thyme oil, natural zeolite, shelf-life extension

## Abstract

This study presents, the development of a green method to produce rich in thymol natural zeolite (TO@NZ) nanostructures. This material was used to prepare sodium-alginate/glycerol/xTO@NZ (ALG/G/TO@NZ) nanocomposite active films for the packaging of soft cheese to extend its shelf-life. Differential scanning calorimetry (DSC), X-ray analysis (XRD), scanning electron microscopy (SEM), and Fourier-transform infrared spectroscopy (FTIR) instruments were used for the characterization of such nanostructures and films, to identify the thymol adsorbed amount, to investigate the thermal behaviour, and to confirm the dispersion of nanostructure powder into the polymer matrix. Water vapor transmission rate, oxygen permeation analyzer, tensile measurements, antioxidant measurements, and antimicrobial measurements were used to estimate the film’s water and oxygen barrier, mechanical properties, nanostructure’s nanoreinforcement activity, antioxidant and antimicrobial activity. The findings from the study revealed that ALG/G/TO@NZ nanocomposite film could be used as an active packaging film for foods with enhanced, mechanical properties, oxygen and water barrier, antioxidant and antimicrobial activity, and it is capable of extending food shelf-life.

## 1. Introduction

In the last few years, petroleum-based food packaging materials have been replaced with biodegradable biopolymers as a result of circular economy and sustainability [1,2,3,4,5]. Thus, the utilization of protein and polysaccharide-based biopolymer hybrid nanostructure materials in the food industry has been increased due to their non-toxicity, biodegradability, ability to form gels, encapsulate and deliver bioactive compounds such as essential oils [6,7,8]. Such novel biopolymer-based food packaging systems are suitable for active food packaging applications. Active food packaging is defined as “packaging in which subsidiary constituents have been deliberately included in or on either the packaging material or the package headspace to enhance the performance of the package system” [4].

Alginate (ALG) has become one of the most popular natural polysaccharides extensively used in the development of delivery systems for food bioactive ingredients. The suitability of this material for such purposes is due to its ionic crosslinking ability, pH responsiveness, excellent biocompatibility, biodegradability, and low price [5]. Alginate (ALG) is an unbranched anionic polysaccharide consisting of β-D-mannuronic acid (M) and α-L-guluronic acid (G) linked by glycosidic bonds. The structure of alginate depends primarily on the monomer composition, the M/G ratio, the polymer sequence, and the molecular weight of the linear chain. The structure of alginate and the M/G ratio are crucial for its capability to deliver bioactive compounds. For example, a higher concentration of G blocks generates more rigid hydrogels with larger pores [6], which leads to the easier release of immobilized bioactive components from the polymer matrix. On the contrary, higher M block content is more suitable for the formulation of softer edible films and coatings with lower gas permeability [7,8].

Under the same spirit, there is a trend to replace the “commonly” used antioxidant and/or antimicrobial chemical agents such as Butylated hydroxytoluene (BHT) and Butylated Hydroxyanisole (BHA) which are added directly to the food. The use of such chemicals was replaced with the use of essential oils [9,10] or other bioactive phytochemicals [11] in active packaging film gels, and coating is taking place. Essential oil loss due to evaporation phenomena was reduced using various nanomaterials as nanocarriers in food delivery systems and active food packaging applications. Such materials were developed based on the food nanotechnology concept [12,13,14]. Nanoclays, such as montmorillonite [15,16,17] and halloysite [18,19,20] were used both as reinforcements and as essential oil nanocarriers for controlled released applications [21]. These nanoclay based essential oil nanocarriers were incorporated into polymer [21,22,23] or biopolymer [16] networks. The new composite materials were promising for active packaging film applications with antioxidant and/or antimicrobial activity. Natural zeolite (NZ) is another, abundant nanomaterial promising for food preservation and food packaging applications [24] as is reported in the literature [25,26,27]. Rešček et al., 2018 [28] developed double-layered polyethylene/caprolactone packaging films modified with zeolite and magnetite. It was shown that the addition of zeolite improved the mechanical and barrier properties of obtained films. Youssef et al., 2019 [29] prepared carboxymethyl cellulose/polyvinyl alcohol films modified with zeolite which was firstly doped with Ag and Au ions. It was shown that the addition of this modified zeolite enhanced the mechanical, barrier and antimicrobial properties of the obtained packaging films. Recently, Nascimento Souza et al., 2020 [30] prepared chitosan packaging film and used zeolite as an ethylene scavenger. To the best of our knowledge, there is no study on the use of natural zeolite (NZ) as nanoreinforcement and/or essential oil nanocarrier in ALG based film preparation.

Cottage cheese is a highly consumed type of cheese which, however, is easily acidified. Because of its high moisture content i.e., about 75%, and pH values over 4.5, the shelf-life of this product is restricted to 15 days [31]. It is well documented that various types of spoilage bacteria, yeasts, and molds that may develop on the cheese surface during storage can influence the shelf life of cheese, particularly in the case of soft and spread cheese. To prevent damage and spoilage, soft and fresh cheese are currently packaged with active cheese technology [32].

In this study natural zeolite (NZ) was firstly modified with thyme essential oil (TO) and produced a novel TO@NZ hybrid nanostructure. These nanostructures were characterized with XRD analysis and FTIR spectrometry. They were directly added to sodium alginate (ALG) plasticized with glycerol (G) hydrogels and produced novel ALG/G/TO@NZ active packaging films. The TO@NZ hybrid nanostructure content was fixed to 5, 10, and 15% wt. The properties of these films were compared with the properties of films prepared with pure NZ. The obtained ALG/G/NZ and ALG/G/TO@NZ films were also characterized with XRD analysis and FTIR spectrometry. Moreover, they were tested for their mechanical and water/oxygen barrier properties. The antioxidant and antimicrobial capacity of the obtained films was also evaluated. Finally, the most active films were used as packaging films to extend the shelf-life of soft cheese. The innovation of the current study can be summarized in the following three points: (1) modification/preparation of a rich in thymol content NZ nanostructure via a green evaporation/adsorption method, (2) development of edible active packaging films based on an ALG/G biopolymer matrix by using the novel TO@NZ nanostructure, and (3) use of such edible active packaging films to extend the self–life of a soft cottage-cheese.

## 2. Results and Discussion

### 2.1. GC-MS Results

The results of the GC-MS analysis of the TO as received and of the remaining after the first stage distillation process TO are summarized in Tables S1 and S2. The main substances of the TO as received are p-cymene 12.3%, D-limonene 16.5%, and thymol 56.7% (see Table S1). At the end of the first stage distillation process, the remaining TO does not contain p-cymene and D-limonene and contains 86.7% thymol (see Table S2).

### 2.2. DSC Results

**Figure 1:**
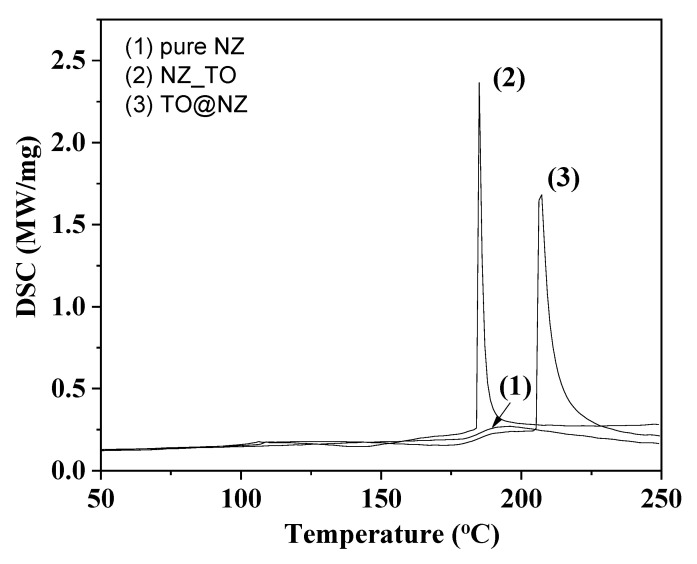
DSC plots of (1) pure NZ, (2) TO_NZ hybrid nanostructure and (3) TO@NZ hybrid nanostructure.

Figure 1 presents DSC plots of pure NZ, TO@NZ, and TO_NZ hybrid nanostructures in the range of 50–250 °C.

All curves exhibit a small, wide, and broad peak starting at around above 100 °C. This peak is attributed to the exothermic water evaporation process. In the case of TO_NZ and TO@NZ hybrid nanostructures two sharp exothermic peaks at approximately 185 °C and 230 °C are observed correspondingly. Additionally, in the case of TO_NZ hybrid nanostructure, there is also a wide broad peak starting above 200 °C. Precisely in the case of TO_NZ hybrid nanostructure the sharp peak at 185 °C does not ends and continues above 200 °C. This peak at 185 °C corresponds to p-Cymene and D-Limonene molecules’ evaporation while the peak at 230 °C corresponds to the thymol molecules’ evaporation [33,34,35]. This observation indicates that molecules that existed in the TO_NZ hybrid nanostructure are of different kinds of molecules that existed in the TO@NZ hybrid nanostructure. In the case of TO_NZ hybrid nanostructures, Limonene, Cymene, and Thymol molecules were adsorbed while in the case of TO@NZ hybrid nanostructures the Limonene and Cymene were removed from TO during the distillation process, and thymol was mainly adsorbed. Additionally, in the case of TO_NZ hybrid nanostructures, higher amounts of Limonene and Cymene molecules were adsorbed.

The DSC results indicate that the TO evaporation process led to the adsorption of higher quantities of D-Limonene and p-Cymene than thymol on the NZ surface (TO_NZ hybrid nanostructure). This happens probably because of their lower evaporation temperature. On the contrary, when D-Limonene and p-Cymene molecules were removed from TO via the distillation process, thymol is the main substance which was adsorbed on the NZ surface (TO@NZ hybrid nanostructure). In other words, from DSC plots, it is clear that the TO_NZ hybrid nanostructure was rich in D-Limonene and p-Cymene molecules while TO@NZ was rich in thymol molecules.

### 2.3. XRD Analysis

**Figure 2:**
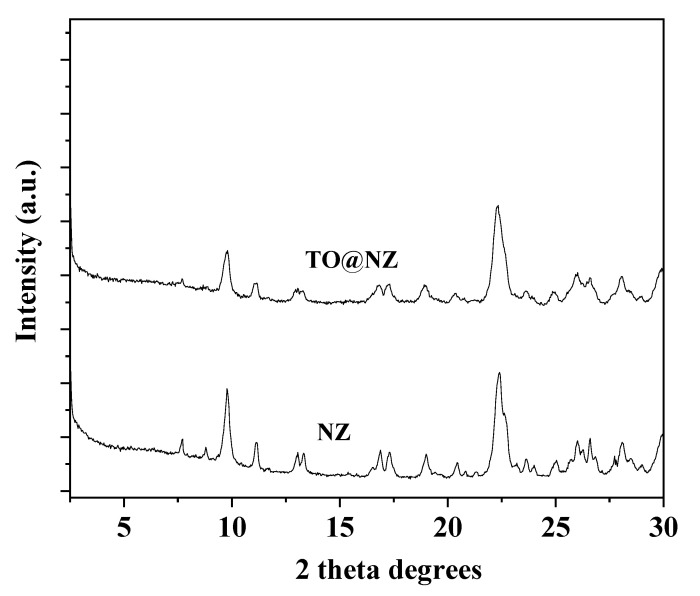
XRD plots of as received NZ and TO@NZ hybrid nanostructure.

The XRD plots of the as received NZ and of the modified TO@NZ hybrid nanostructure are presented in Figure 2.

The observed reflections in patterns of both NZ and TO@NZ materials are attributed to Heulandite Ca(Si_7_Al_2_)O_16 ×_ 6H_2_O monoclinic crystal phase (PDF-41-1357). This means that the adsorption of TO into NZ did not affect the crystal phase.

**Figure 3:**
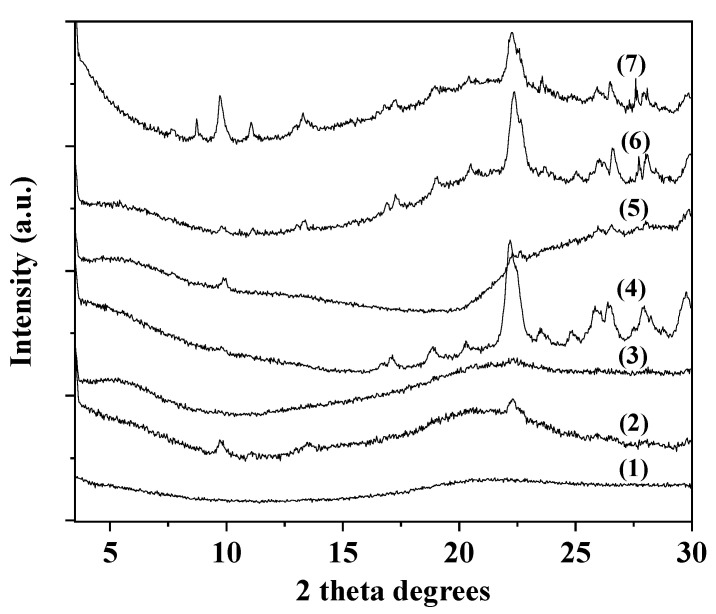
XRD plots of: (1) ALG/G, (2) ALG/G/5NZ, (3) ALG/G/10NZ, (4) ALG/G/15NZ, (5) ALG/G/5TO@NZ, (6) ALG/G/10TO@NZ AND (7) ALG/G/15TO@NZ obtained films.

The XRD plots of pure ALG/G, ALG/G/xNZ, and ALG/G/xTO@NZ nanocomposite films are presented in Figure 3 (where x is the nanostructure composition).

Unplasticized alginate films exhibited two broad peaks with central positions at 2θ = 13.5° and 21.6° [36,37]. As it is observed in Figure 3, in the case of such ALG/G films the peak at 2θ = 13.5° disappeared indicating a lower proportion of the amorphous structure with larger chain distances. This is a result of water and glycerol de-structuration [36]. No changes of the ALG crystallinity were observed with the addition of either NZ or TO@NZ hybrid nanostructures. Moreover, after an initial additive loading into the polymeric matrix, as the % wt. content of NZ or TO@NZ hybrid nanostructure increases the reflections of zeolite’s crystal phase are increase. This indicates that the higher dispersion of such materials in the ALG/G film is obtained only for low % wt. loadings i.e., <10% wt.

### 2.4. FTIR Spectroscopy

**Figure 4:**
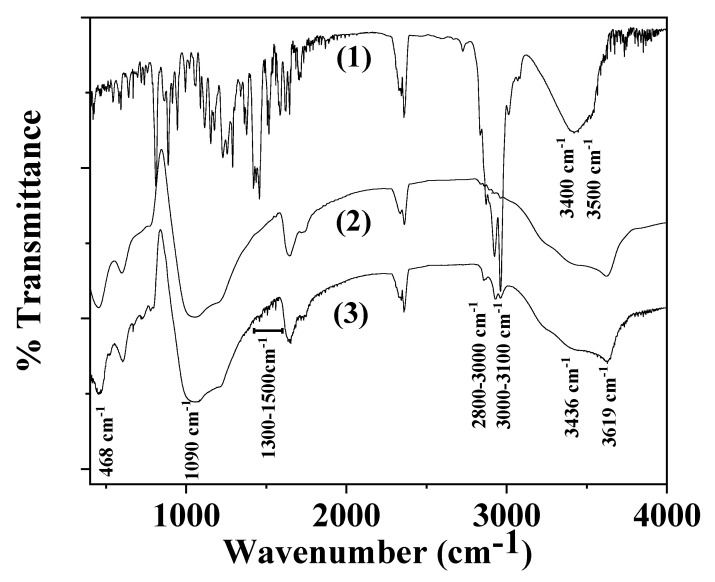
FTIR plots of: (1) TO as received, (2) NZ as received, (3) modified TO@NZ hybrid nanostructure.

Line (1) in Figure 4 represents the FTIR spectra of the as received TO. In the same figure, Line (2) is assigned to the as received natural zeolite FTIR spectra, and Line (3) to the modified rich in thymol natural zeolite TO@NZ.

In the FTIR plot of the TO material, i.e., Line (1) in Figure 4, the bands at ~3400 cm^−1^ and at ~3500 cm^−1^ are assigned to hydrogen-bonded OH stretching, and the bands at ~3100–3000 cm^−1^ are devoted to aromatic and alkenic C-H=C-H stretch vibrations. Three more bands are observed between 2800 and 3000 cm^−1^ which are attributed to the C-H stretch vibration of aliphatic CH_2_ bonds. These bands are the strongest evidence that TO was adsorbed on NZ substrate because they are not covered by pristine NZ bands. For wavenumbers between 1500 cm^−1^ and 1300 cm^−1^ as well as for lower than 1000 cm^−1,^ there are several bands assigned to TO which are attributed to the C-H bending of the aliphatic CH_2_ groups and C-O-H bending. These bands do not be overlapped with those of NZ. spectra and thus they could be visible in an NZ spectrum with adsorbed TO [15,38].

In the FTIR plots of NZ and TO@NZ powders ((see line (2) and line (3) in Figure 4), the bands at 3619 and 3436 cm^−1^ are assigned to the OH group stretching mode. The band at 1650 cm^−1^ corresponds to the OH group bending mode. The band at 1090 cm^−1^ to the Si-O stretching vibration and at 468 cm^−1^ to the -SiO_4_- bending mode [39,40,41]. It is obvious from the FTIR plot of TO@NZ that characteristic bands of TO exist in the range of 2800–3100 cm^−1^, 1300–1500 cm^−1^, and 500–1000 cm^−1^. This indicates that the adsorption of TO molecules in the NZ occurs. The absence of band shift between NZ and TO@NZ plots means that the adsorption process is rather a physisorption than chemisorption.

**Figure 5:**
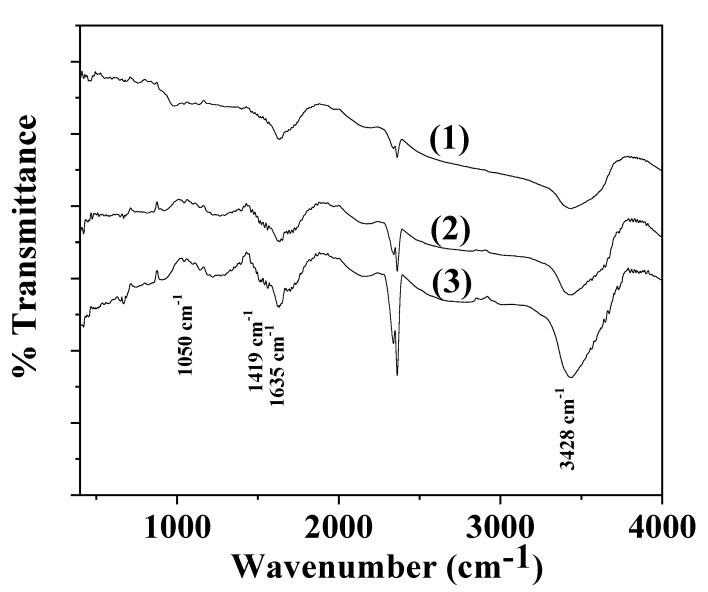
Representative FTIR spectra of (1) ALG/G, (2) ALG/G/NZ, (3) ALG/G/TO@NZ obtained films.

Line (1) in Figure 5 depicts the FTIR plots of ALG/G while Line (2) shows the FTIR spectra of ALG/G/NZ material, and Line (3) of ALG/G/TO@NZ films.

Line 1 of Figure 5 corresponds to the FTIR plot of pure ALG/G film while line 2 is a representative FTIR plot of ALG/G/10NZ. Finally, line 3 is assigned to FTIR measurements of ALG/G/10TO@NZ nanocomposite films. The characteristic sodium-alginate peaks are observed in all plots. A broad band at 3.428 cm^−1^ is assigned to hydrogen-bonded O–H stretching vibrations [42]. The band at 1635 cm^−1^ is attributed to the asymmetric stretching vibration of COO groups, the band at 1419 cm^−1^ to the symmetric stretching vibration of COO groups, and the band at 1050 cm^−1^ to the elongation of C-O groups [43]. It is obvious from lines (2) and (3) that the addition of NZ and TO@NZ hybrid nanostructures causes an increase to the bands at 3.428 cm^−1^ and 1635 cm^−1^ which could be attributed to strong interactions of ALG chains with NZ and TO@NZ hybrid nanostructures. This interaction is higher in the case of the TO@NZ hybrid nanostructure. Thus, it is revealed that modified TO@NZ hybrid structure interacts better with ALG/G matrix compared to the relevant of the pure NZ material. Furthermore, the absence of TO peaks in ALG/G/10TO@NZ spectra indicates that the TO molecules are not in the surface but in the inner area of the ALG/G matrix and supports the relaxation between the NZ material and the ALG/G matrix.

### 2.5. SEM Images

A SEM instrument equipped with an EDS detector was used to investigate the surface/cross-section morphology of the pure ALG/G film as well as of the ALG/G/xNZ and ALG/G/xTO@NZ hybrid nanocomposite films. The results confirmed that the NZ and the TO@NZ hybrid nanostructures were homogeneously dispersed in the ALG/G polymeric matrix. The chemical elements contained in the pure and final nanocomposite active packaging films were identified by carrying out EDS analysis on the surface of the materials.

**Figure 6:**
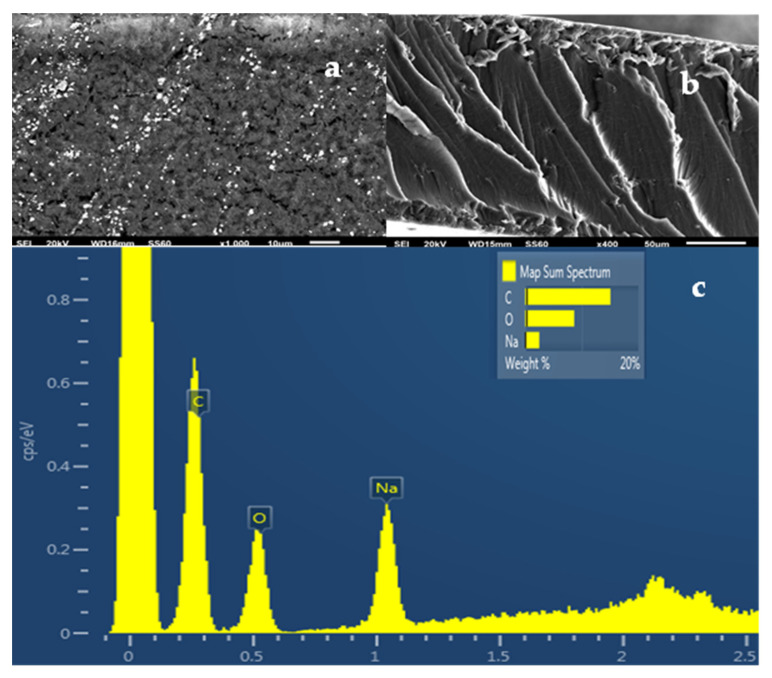
(**a**) SEM images of the surface and (**b**) cross-section for the pure film of ALG/G. (**c**) Energy dispersive spectrometer (EDS) spectrum and relative elemental analysis of the surface (inset) from the SEM image (**a**).

**Figure 7:**
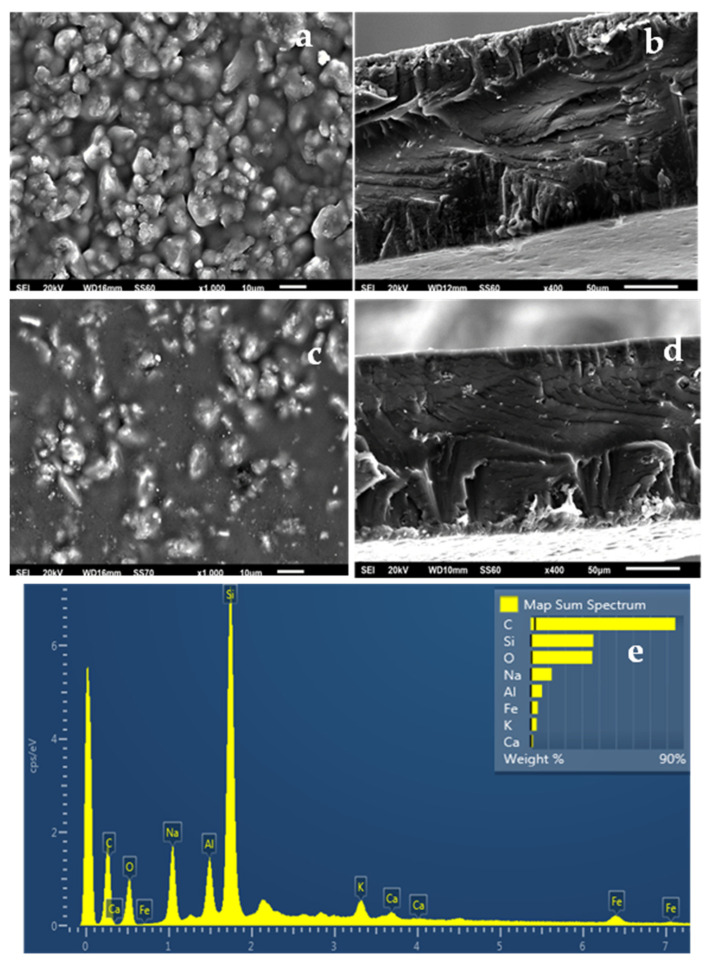
(**a**,**c**) SEM images of the surface and (**b**,**d**) cross-section for the nanocomposite films of ALG/G/5NZ (**a**,**b**) and ALG/G/5TO@NZ (**c**,**d**) respectively. (**e**) Energy dispersive spectrometer (EDS) spectrum and relative elemental analysis of the surface (inset) from the SEM image (**a**).

**Figure 8:**
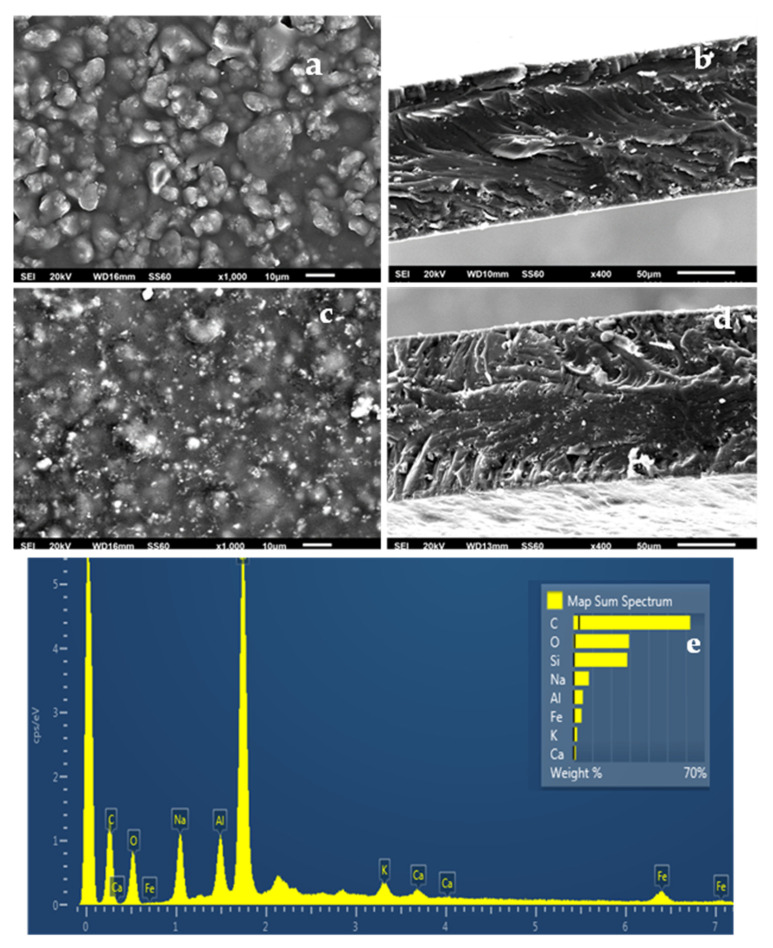
(**a**,**c**) SEM images of surface and (**b**,**d**) cross-section for the nanocomposite films of ALG/G/10NZ (**a**,**b**) and (**c**,**d**) ALG/G/10TO@NZ (**c**,**d**) respectively. (**e**) Energy dispersive spectrometer (EDS) spectrum and relative elemental analysis of the surface (inset) from the SEM image (**a**).

**Figure 9:**
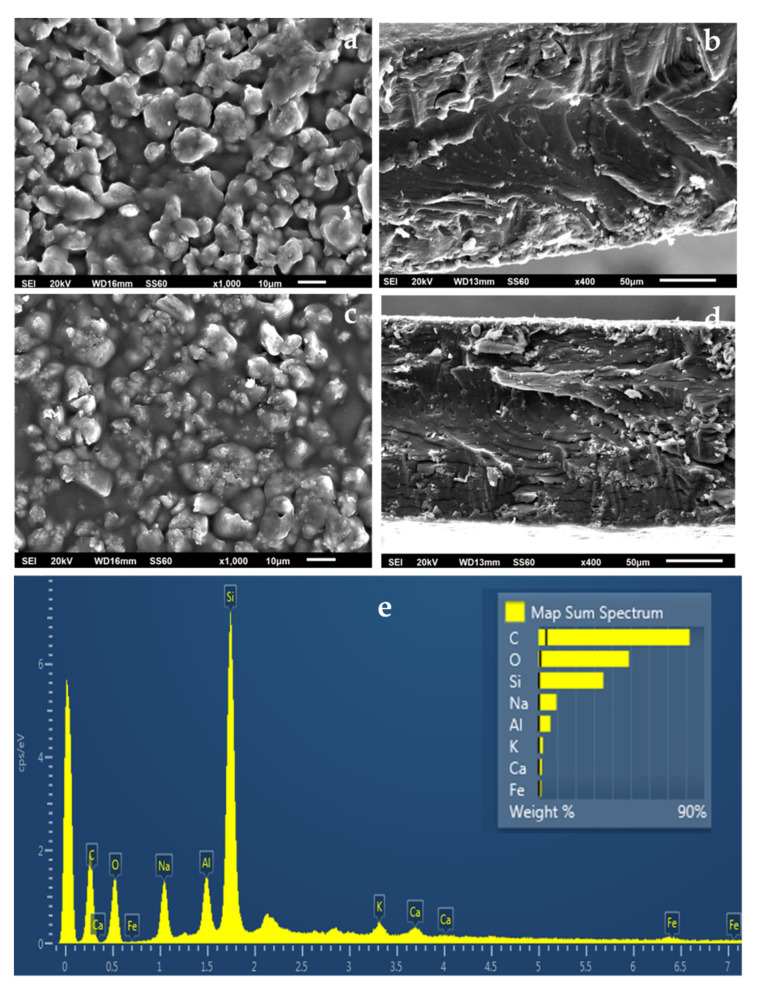
(**a**,**c**) SEM images of surface and (**b**,**d**) cross-section for the nanocomposite films of ALG/G/15NZ (**a**,**c**) and ALG/G/15TO@NZ (**c**,**d**) respectively. (**e**) Energy dispersive spectrometer (EDS) spectrum and relative elemental analysis of the surface (inset) from the SEM image (**a**).

The SEM images in Figure 6a,b show the expected smooth morphology inside and outside of the neat ALG/G polymer matrix. The EDS spectra in Figure 6c certify the existence of carbon (C), oxygen (O), and sodium (Na) on the surface of such films which is expected because of the sodium alginate. Figure 7e, Figure 8e and Figure 9e show EDS chemical analysis of nanocomposite active packaging films with different concentrations of pure NZ and TO@NZ hybrid nanostructure i.e., 5, 10, and 15% wt. In addition to the above mentioned presence of (C), (O), and (Na), the presence of typical elements, such as Si, Al, Fe, K, and Ca, confirm the existence of NZ and TO@NZ in such nanocomposite films. Moreover, the increase of (Na) content of the ALG/G/xNZ films i.e., ~10% compared to the relevant content of the pure ALG/G films i.e., ~2% indicates the incorporation of the natural zeolite into the polymer matrix. Surface and relative cross-section images of ALG/G/xNZ and ALG/G/xTO@NZ with different ratios x of NZ and TO@NZ are presented in Figure 7, Figure 8 and Figure 9.

It is obvious from Figure 7, Figure 8 and Figure 9 that after the incorporation into the polymer matrix, the increase of the content of NZ or TO@NZ nanocomposite material caused an increase to the aggregation degree. Nevertheless, SEM images of the final nanocomposite films show that the nanohybrids were homogeneously dispersed, which indicates their enhanced compatibility with the polymer matrix. Moreover, SEM surface and cross-section images were shown more homogenous dispersion in the case of TO@NZ hybrid nanostructure in nanocomposite films compared to the relevant of pure NZ. This means that the TO@NZ hybrid nanostructure was incorporated significantly better in the polymer matrix compared to the incorporation of the respective pure NZ.

### 2.6. Tensile Properties

The calculated values of elastic modulus (E), ultimate strength (σ_uts_), and elongation at break (%ε) for all ALG/G/xNZ and ALG/G/xTO@NZ nanocomposite films are listed in Table 1.

It is obvious from Table 1 that the addition of both NZ and TO@NZ hybrid nanostructure increases stiffness and strength and decreases %elongation at break values. The nanocomposite film with the higher strength was the ALG/G/15NZ and ALG/G/15TO@NZ. This result is in accordance with previous reports where zeolite was successfully incorporated into polyethylene/caprolactone [28], cellulose [29,44], and chitosan [30] films as nano-reinforcement. The result also agrees with the FTIR morphological evaluation of such films where an interplay between NZ, TO@NZ hybrid nanostructures and ALG/G matrix was obtained. In general, ALG/G/TO@NZ based nanocomposite films exhibited higher elongation at break values than the ALG/G/NZ due to the presence of TO molecules which acted as plasticizers [22,45].

### 2.7. UV-vis Transmittance of Films

**Figure 10:**
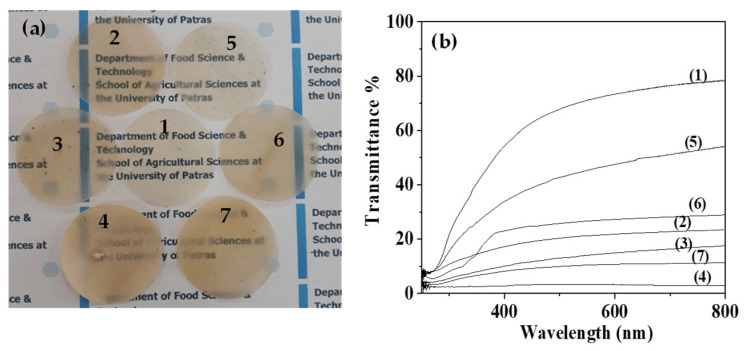
(**a**) photo images of all prepared films, (**b**) UV-vis transmittance of all prepared films. (1) pure ALG/G, (2) ALG/G/5NZ, (3) ALG/G/10NZ, (4) ALG/G/15NZ, (5) ALG/G/5TO@NZ, (6) ALG/G/10TO@NZ and (7) ALG/G/15TO@NZ films.

Figure 10a presents the images of as prepared pure ALG/G film as well as of ALG/G/xNZ and ALG/G/xTO@NZ nanocomposite films. Figure 8b presents the UV-vis transmittance plots of all obtained films. It is obvious from both images and UV-vis plots that the TO@NZ based films are more transparent than NZ based counterparts. Higher transparency indicates higher nanofiller dispersion and integration inside the polymer matrix. Thus, it seems that the TO enhances the dispersion of the NZ in ALG/G/xTO@NZ nanocomposite films. Moreover, the lowest transparency is obtained for ALG/G/15NZ and ALG/G/15TO@NZ films.

### 2.8. Water-Oxygen Barrier Properties

Water vapor transmission rate (WVTR) and oxygen transmission rate (OTR) values for all ALG/G/xNZ and ALG/G/xTO@NZ films are listed in Table 2. Using these values, water vapor diffusivity (D_WV_) and oxygen permeability (Pe_O2_) values were calculated and listed in the same table.

Observing the water diffusivity values, we can conclude that the addition of NZ initially caused a reduction to water diffusivity i.e., a minimum value of 3.97 cm^2^/s to the content of 10% wt. Beyond that, the water vapor diffusivity starts to increase and, for NZ content 15% wt., its value became almost equal to the relevant of the initial raw material. In the case of TO@NZ hybrid nanostructure the minimum water diffusivity value observed for 5% wt. TO@NZ content and for 15% wt. content the water vapor diffusivity was higher than the relevant of the initial raw material. In general, 10% wt. NZ content in the polymer matrix exhibits a water vapor barrier almost equal to the relevant of 5% wt. TO@NZ content and lower enough compared to the relevant of the initial raw material. Beyond these concentrations, the extra addition of such nanostructured materials to the polymer matrix caused an increase to the water vapor diffusivity. Concerning the oxygen permeability, the minimum coefficient values compared to the relevant of the initial raw material were observed for 5% wt. NZ content and for 10 %wt. TO@NZ content. Increasing these concentrations, the oxygen permeability starts to increase. Considering Table 2 and according to the previous observations, we could say that the optimum NZ or TO@NZ additive concentration for the highest water vapor or oxygen barrier lies in the range of 5–10% wt.

### 2.9. Antioxidant Activity of Films

Antioxidant activity values of all the tested films were measured following the diphenyl-1-picrylhydrazyl (DPPH) assay method and are listed in Table 3.

It is already known that Sodium alginate exhibits antioxidant activity [46,47]. According to our measurements, the antioxidant activity of the pure ALG/G film was 8.7%. In the case of NZ incorporation into the polymer matrix, this activity was increased by the addition of 10 %wt. material and over. According to literature, NZ acts as an antioxidant agent due to the entrapment of free radicals in its porous [48]. The microporosity presence in the NZ pore structure enhances the adsorption properties of this material and consequently the antioxidant activity [49]. Thus, the increased antioxidant activity of the NZ containing films measured in this study could be attributed to the adsorption of DPPH ions and free radicals in its structure. According to Table 3 values, even the lower TO@NZ load exhibits stronger antioxidant activity compared to the relevant value of the film with the higher NZ content. This happens because essential oils and especially TO exhibit significant antioxidant activity [50]. As an overall conclusion, we could say that, based on Table 3 values, ALG/G/15TO@NZ films show the highest antioxidant activity.

### 2.10. Antimicrobial Tests

#### 2.10.1. MICs and MBCs Determination of TO@NZ Hybrid Nanostructure against LAB and Pathogen Bacteria

The MICs and MBCs of TO@NZ against different bacteria are presented in Table 4.

TO@NZ hybrid nanostructure exerted antimicrobial activity against all tested bacteria for at least 2 days, although its effectiveness was dependent on the targeted strain. It was observed that both the lactic acid bacteria were inhibited at 0.025% concentration, which was the lowest MIC compared with that of the pathogenic strains. However, the MICs measured for the pathogenic strains were two times higher (0.05%) for *S. aureus* and 20 times higher (0.1) for *L. monocytogenes* and *Enterococcus faecalis* strains. These results indicate that the hybrid nanostructure can inactivate LAB and presumptive pathogenic microorganisms associated with spoilage and the safety of dairy products. Similar results have been reported [51] for the antimicrobial activity of thyme essential oil in Coahlo fresh cheese, where the MIC of thyme oil on pathogenic *S. aureus* and *L. monocytogenes* was two times higher (2.5 μL/mL) than that (1.25 μL/mL) for starters *L. lactis* ssp. *lactis* and *L. lactis* ssp. *cremoris.* These results suggest that the doses of EO thyme alone and in the form of TO@NZ hybrid nanostructure to control pathogenic bacteria should affect the growth and survival of starter cultures. On the other hand, carvacrol and thymol have been reported as the most inhibitory essential oils against non-starter lactic starter bacteria (NSLAB) with MICs of 0.1% (*w*/*v*) [52]. Reduction of 2-log CFU/mL against *L. buchneri* and *P. acidilactici* was achieved for thymol 0.1% (*w*/*v*) while this concentration was bactericidal against *L. citrovorum* (>4-log reduction). These results indicate that thymol oil can inactivate the non-starter LAB. According to the common opinion, such bacteria cause spoilage of shelf-stable low-acid dairy products. In particular, antimicrobial activity of thyme oil on milk and dairy products was established by previous studies [53,54].

#### 2.10.2. Antimicrobial Activity of Active Films Application on Cheese against *S. aureus*

**Figure 11:**
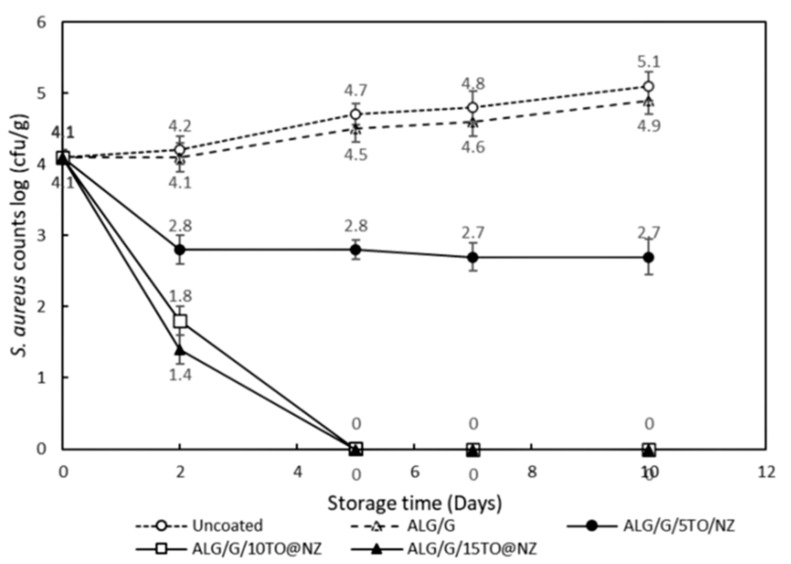
Changes in *Staphylococcus aureus* ATCC1534 counts in cottage cheese samples wrapped with alginate-based edible films (ALG/G/xTO@NZ) with the hybrid nanostructure TO@NZ in different concentrations.

The results for the antimicrobial activity of hybrid nanostructured films used for packaging of cottage cheese under 10 °C storage temperature are shown in Figure 11.

Given the strong antibacterial activity of the TO@NZ bioactive hybrid nanostructure against the pathogen strains, the prepared ALG/G/TO@NZ active films were studied for the antimicrobial activity against the pathogenic bacteria *S. aureus* ATCC1538 in cottage fresh cheese, as a frequently associated toxinogenic bacteria with fresh or low-ripened cheeses. During 15 days of storage of cheese at an abuse temperature of 10 °C, it was found that *S. staphylococcus* ATCC1538 levels increased in the control uncoated sample and the sample coated with ALG/G film. However, a significant decrease of this bacterium concentration occurred in all cheese samples coated with ALG/G/xTO@NZ films. Furthermore, the antimicrobial activity was significantly related with films containing essential oils (*p* < 0.05). The initial count of *S. aureus* ATCC1538 in the uncoated control sample was 4.25 log_10_ cfu/g on the 1st day of storage and this value significantly increased (*p* < 0.05) to 4.75 (0.50 log_10_ cfu/g increase) by the 15th day of storage. However, in the case of the coated sample with the ALG/G film without the TO@NZ hybrid nanostructure, there was a slight decrease (not statically significant) of count to 4.1 log_10_ (cfu/g), which shows a bacteriostatic activity on *S. aureus* strain. The application of ALG/G/5TO@NZ active film on cheese resulted in a significant reduction of about 1-log_10_ (cfu/g) (*p* < 0.05) against the *S. aureus* ATCC1538 population from the 2nd day of storage, which remained at this level during the whole storage period (15 days), indicating a bacteriostatic activity. However, in the case of ALG/G/10TO@NZ and ALG/G/15TO@NZ active films application on cheese, a significant decrease of 2 log_10_ (cfu/g) of *S. aureus* population was counted from the 2nd day of storage and from the 5th day no counts detected up to 15th day, indicating a strong bactericidal effect of both active films against the pathogen. Similarly, a significant relationship has been detected between the thyme-fortified edible whey isolate-based films (WPIOF) and *S. aureus* inactivation in Kashar cheese [55]. Opposite of the studies in which the essential oils were added directly to foods, the number of studies in which the essential oils were used as a composite of packaging materials are fewer [56]. As the time passes or/and the temperature increases the possibility for food illness to arise increases, given that an *S. aureus* population of 10^5^ cfu/g consists the threshold for alpha-staphylotoxin production in cheese [56]. In our study, the cheese was strongly protected from Staphylococcal intoxication even under abusing storage temperature of 10 °C. However, we should consider that the application of ALG/G/TO@NZ active films in fermented dairy products (such as cheeses) in doses enough to control pathogenic bacteria could also influence the growth and survival of lactic acid bacteria, which probably affect the post-acidification activity and improve the product’s self-life [57].

## 3. Conclusions

In conclusion, we could say that NZ or TO@NZ nanostructures were incorporated perfectly with the ALG/G polymer matrix and provide a very promising active film for food packaging, which could extend the soft cheese shelf-life. Furthermore, the effort to develop a more environmentally friendly process seems to be successful because the new materials were based on natural raw materials with reduced use of chemicals. The overall success of this study was confirmed by the XRD and FTIR results while FTIR and DSC indicate a film rich in thymol oil physiosorbed in NZ nanocomposite. Consistent with the antimicrobial, antibacterial, and antioxidant measurements, the higher the thymol concentration, the better antimicrobial, antibacterial, and antioxidant results. Actually, there is a threshold of TO@NZ hybrid nanostructure concentration which is required for the film to exhibit inhibition and bactericidal activity against the tested bacteria and pathogens. This also improves the mechanical properties of the film because TO acts as a plasticizer. Nevertheless, in line with SEM and UV-vis results, the increase of TO@NZ concentration led to lower transparency and higher nanostructure aggregation in the film. Thus, there is an optimum for TO@NZ concentration in the polymer matrix and this is in the range between 5% wt and 10% wt. In this range, the D_WV_ and Pe_O2_ coefficients exhibit a local minimum which means the highest water and oxygen barrier.

## 4. Materials and Methods

### 4.1. Materials

Edible Natural Zeolite was purchased by a local pharmacy market. Sodium Alginate was purchased from Acros-Organics (Zeel West Zone 2, Janssen Pharmaceuticalaan 3a B2440 Geel, Belgium). Glycerol was purchased from Carlo-Erba (Denzlinger Str. 27, 79312 Emmendingen, Germany). Thyme oil was purchased from a local pharmacy market and produced by Chemco (Via Achille Grandi, 13–13/A, 42030 Vezzano sul Crostolo RE, Italy). The cottage cheese which used during the experimental process was purchased from a local Greek market with the brand name TYRAS, TYRAS S.A., Trikala, Thessalia, Greece, and immediately transferred to the laboratory refrigerator and stored under a temperature of 4 °C. According to the products specification, the expiration date of the product was 12 days after the production date. Moreover, the product expired in 4 days if the package film opened. Furthermore, the product could be stored for 75 days if it was freezed at temperature below −16°C and with closed packaging film. The PCA was found 7.52 cfu/g, while pH was determined at 4.9.

### 4.2. Thyme oil GC-MS Analysis

An amount of 1 μL thyme oil/pentane dilution with 2 mg/mL concentration was injected into the GC/MS (in splitless mode). The analysis of each sample was carried out three times. The used GC instrument was a Finnigan Trace GC Ultra 2000 equipped with a Finnigan Trace DSQ MSD (Thermo Electron Corporation, Waltham, MA, USA) detector which was operated in EI mode. The conditions of separation process inside the capillary column HP-5MS (Agilent Technologies, USA) (30 m × 0.32 mm, 0.25 μm film thickness) were as follows: carrier gas Helium, flow rate 0.8 mL/min, initial temperature of column 60 °C, and the separation temperature ramp was achieved heating the column to 240 °C at a rate of 3 °C/min for 10 min. The detector voltage and temperature were kept at 70 eV and 250 °C respectively while the injector temperature was adjusted to 200 °C. ADAMS, Wiley275, NIST, and in-house created libraries were used for the compounds’ identification. The method for this identification was by comparing the retention times and the mass spectra of volatiles [58,59].

### 4.3. Preparation of TO@NZ Hybrid Nanostructures

The preparation of TO@NZ bioactive hybrid nanostructures was carried out based on the method reported elsewhere for nanoclays modification [15] with some small changes. In a spherical flask, an amount of 20 mL TO were heated to boil. Before its use, the thyme oil was freed up from the Limonene and Cymene by distillation process at 190 °C. An amount of 6 mL was removed which is approximately 30% of the total amount. This result is in close agreement with the GC measurements data. The distillation process was the first stage of the overall process which was followed in this study. The distillation temperature was chosen a little higher than Limonene’s and Cymene’s boiling point temperatures (170–175 °C) and lower than thymol’s boiling point temperature (230–235 °C). After the first stage, the remaining liquid colour was changed to dark brown and in line with LC-MS/MS measurements, it was rich of thymol oil. In the second step, an evaporation-adsorption process was carried out. The remained liquid from the first stage was boiled up to 250 °C. The produced thymol rich steam passed through a bed of 3 gr NZ which strongly adsorbed the essential oil. The remaining outlet steam was cooled down through a condenser and the final liquid was collected. At the same time, an amount of as received thyme oil was adsorbed by NZ material and reference hybrid nanostructured material TO_NZ was prepared. The %wt. adsorbed TO in both TO@NZ and TO_NZ hybrid nanostructures was calculated gravimetrically and was approx. 80% wt. for both hybrid nanostructures.

### 4.4. Preparation of ALG/G/NZ and ALG/G/TO@NZ Active Films

A quantity of 2 g ALG and of 1 g G was spread into 100 mL of distilled water and heated until a homogeneous hydrogel was obtained. An appropriate amount of NZ or TO@NZ powder was dispersed in 10 mL of distilled water using a glass beaker. Then, the homogeneous ALG/G hydrogel was added gradually in the NZ or TO@NZ suspension and vigorously stirred for 2 h. The obtained ALG/G/NZ and ALG/G/TO@NZ hydrogels were then spread in petri dishes of 11 cm diameter and dried at 25 °C. The obtained films were pilled of and stored further in a desiccator at 25 °C and 50 %RH. In Table 5, the code names and the amounts of ALG, G, NZ, and TO@NZ, which were used to obtain such films, are summarized.

### 4.5. DSC Measurements

A DSC214 Polyma Differential Scanning Calorimeter (NETZSCH manufacturer, Selb, Germany) was used to study the thermal behaviour of the obtained TO@NZ hybrid and of the pure NZ nanostructures. Quantity 1.2–3.3 mg of samples was heated from 50 °C to 300 °C, under nitrogen atmosphere, and an increasing temperature rate of 10 °C/min.

### 4.6. XRD Analysis

An Brüker D8 Advance X-ray diffractometer instrument (Brüker, Analytical Instruments, S.A., Athens, Greece) was used for XRD measurements on ALG/G, ALG/G/NZ, and ALG/G/TO@NZ active films, as well as on NZ and TO@NZ hybrid nanostructure powders. The instrument was equipped with a LINXEYE XE High-Resolution Energy-Dispersive detector, and the measurements were carried out in the range 2θ = 0.5–30° and with an increment of 0.03.

### 4.7. FTIR Spectrometry

The chemical structure of both the NZ as received and the modified TO@NZ hybrid nanostructure, as well as of the obtained ALG/G, ALG/G/xNZ and ALG/G/xTO@NZ films, was analyzed with IR spectra measurements. An FT/IR-6000 JASCO Fourier transform spectrometer (JASCO, Interlab, S.A., Athens, Greece) was used and the measurements were carried out in the frequency range of 4000–400 cm^−1^. The measured infrared (FT-IR) spectra resulted from 32 scans with 2 cm^−1^ resolution.

### 4.8. SEM Images

The surface morphology characterization of the obtained films was performed with SEM images. For SEM image measurements a JEOL JSM-6510 LV SEM Microscope (Ltd., Tokyo, Japan) was used equipped with an X-Act EDS-detector by Oxford Instruments, Abingdon, Oxfordshire, UK (an acceleration voltage of 20 kV was applied).

### 4.9. Tensile Properties

Tensile measurements of all prepared ALG/G, ALG/G/xNZ and ALG/G/xTO@NZ films were carried out using a SimantzüAX-G 5kNt instrument (Simandzu. Asteriadis, S.A., Athens, Greece) and as reported by the ASTM D638 method. Three to five samples of each film were used for statistical analysis of tensile properties. The samples, with a shape of dumb-bell and gauge dimensions of 10 × 3 × 0.22 mm, were tensioned at an across head speed of 2 mm/min. Stress, stain, and modulus of elasticity values were calculated using force (N) and deformation (mm) measurements, and the gauge dimensions.

### 4.10. UV-vis Transmittance of Films

Absorbance properties of ALG/G, ALG/G/xNZ, and ALG/G/xTO@NZ films were estimated via UV–vis measurements using a Shimatzu 1900 spectrophotometer. Measurements were carried out in the range of 200 to 800 nm.

### 4.11. Water Vapor Diffusivity

ASTM E96/E 96M-05 method was followed to estimate the Water-Vapor Transmission Rate (WVTR) of ALG/G/xNZ and ALG/G/xTO@NZ films The used apparatus was handmade, and the experiments were carried out at 38 °C and 95% RH according to literature [16,23,60,61]. Film samples was of 2.5 cm diameter and 0.09 mm average thickness and placed on the top of an one-open end cylindrical tube made of plexiglass. The cylinder, which contained dried silica gel inside, was sealed by a rubber O-ring. The test tube was placed in a glass desiccator under an environment of 95% relative humidity (RH) at 38 °C. Such conditions were obtained placing 200 mL of saturated magnesium nitrate solution in this desiccator. Each tube was weighed periodically for 24 h. The WVTR (g∙cm^−2^∙s^−1^) values was calculated using such weighting measurements and according to Equations (1) and (2):

(1) $$\bar{\left(\frac{\Delta G}{\Delta t}\right)}=\frac{1}{n}\overset{n-1}{\underset{i=1}{\sum }}\left(\frac{G_{i+1}-G_{i}}{t_{i+1}-t_{i}}\right)$$

(2) $$\mathrm{WVTR}=\bar{\left(\frac{\Delta G}{\Delta t}\right)}\cdot \frac{1}{A}$$

where: $\bar{\left(\frac{\Delta G}{\Delta t}\right)}$(g∙s^−1^) is the mean water vapor mass diffusion rate for the overall experiment duration, G_i_ (g) is the device weight which balanced at the elapsed time t_i_ (s) from the experiment start, $\frac{G_{i+1}-G_{i}}{t_{i+1}-t_{i}}$ (g∙s^−1^) is the water vapour mass diffusion rate for each time interval, (n) is the number of weight measurements, and A (cm^2^) is the permeation area of the film. Additionally, the tested films were weighed before and after the WVTR test to exclude any absorption phenomena of humidity in the film. ΔG/Δt (g/s) is the water transmission rate through the film which is calculated by the experimental points (G_i_, t_i_).

WVTR is equal to the specific mass flow rate for the diffusion process through a membrane which could be calculated using Fick’s law [62]:

(3) $$\frac{J}{A}=D\cdot \frac{\Delta C}{\Delta x}$$

where J (g/s) is the mass flow rate of a component through the membrane, A (cm^2^) is the membrane cross-sectional area permeated by this component, ΔC (g∙cm^−3^) is the concentration gradient of this component on the two sides of the membrane, and Δx (cm) is the membrane thickness. the humidity concentration in the outer side of the cylinder is 4.36509 × 10^−5^ g∙cm^−3^ (95% RH at 38 °C) and in the opposite side of the film the silica gel absorbs the 100% of the permeated water vapor, which agrees with the ASTM E96/E 96M-05 method, then ΔC = 4.36509 × 10^−5^ g∙cm^−3^. Considering that WVTR = J/A we can calculate the diffusion coefficient D (cm^2^∙s^−1^) for every film via the combination of Equations (2) and (3) which leads to Equation (4):

(4) $$D_{\mathrm{WV}}=\mathrm{WVTR}\cdot \frac{\Delta x}{\Delta C}$$

where WVTR [g∙cm^−2^∙s^−1^)] is the water-vapour transmission rate, Δx (cm) is the film thickness, and ΔC (g∙cm^−3^) is the humidity concentration gradient on the two opposite sides of the film.

### 4.12. Oxygen Permeability

An oxygen permeation analyzer (8001, Systech Illinois Instruments Co., Johnsburg, IL, USA) was used to measure the oxygen transmission rate (OTR) through the tested films. Following the ASTM D 3985 method the experimental conditions were regulated at 23 °C and 0% RH and the measured OTR values were expressed in cc O_2_/m^2^/day.

Gas permeability through polymers can be expressed according to the literature [63], as follows:

(5) $$\frac{J}{A}={\mathrm{Pe}}_{\mathrm{gas}}\cdot \frac{\Delta C}{\Delta x}$$

where J/A (mol∙cm^−2^∙s^−1^) is the specific molar rate of gas which permeates through the membrane, Pe_gas_ (cm^2^∙s^−1^) is the permeability coefficient of the gas, ΔC (mol∙cm^−3^ STP) is the concentration gradient between the two opposite sides of the membrane, and Δx (cm) is the membrane thickness.

Rearranging Equation (5) we can express the permeability coefficient as follows:

(6) $${\mathrm{Pe}}_{\mathrm{gas}}=\frac{J}{A\cdot \Delta C}\cdot \Delta x$$

By carrying out a dimensional analysis, the term J/(A*ΔC) (cm^3^ STP∙cm^−2^∙s^−1^)) is equal to the OTR measurements. Thus, in our case of oxygen gas,

(7) $${\mathrm{Pe}}_{O_{2}}=\mathrm{OTR}\cdot \Delta x$$

where Pe_O2_ (cm^2^∙s^−1^) is the oxygen permeability coefficient, OTR (cm^3^ STP∙cm^−2^∙s^−1^) is the experimentally measured by the instrument oxygen transmission rate, and Δx (cm) is the mean film thickness.

Thus, the oxygen permeability coefficient values (Pe_O2_) of the tested samples were calculated by multiplying the OTR values by the average film thickness which was 0.035 mm. The OTR value for each kind of film was the mean value of measurements on three different pieces.

### 4.13. Antioxidant Activity

To evaluate the antioxidant activity of obtained ALG/G, ALG/G/xNZ, and ALG/G/xTO@NZ films, 300 mg of each film were cut into small pieces and placed inside dark glass bottles with 10 mL of a 40 ppm ethanolic solution of diphenyl-1-picrylhydrazyl (DPPH). A dark glass bottle with 10 mL of ethanolic DPPH solution without the addition of any film was used as the reference sample. The absorbance at 517 nm wavelength of the DPPH solution was measured at 0 h and after 24 h incubation using a Jasco V-530 UV-vis spectrophotometer. For each kind of film, three different samples were measured and the statistical mean was achieved as the final measurement.

The % antioxidant activity after 24 h incubation of films was calculated according to the following equation:

(8) $$\%\;\mathrm{Antioxidant}\;\mathrm{activity}=\frac{{\mathrm{Abs}}_{\mathrm{ref}.}-{\mathrm{Abs}}_{\mathrm{sample}}}{{\mathrm{Abs}}_{\mathrm{ref}.}}\times 100$$

### 4.14. Antimicrobial Activity Tests

#### 4.14.1. Antimicrobial Activity of TO@NZ Bioactive Hybrid Nanostructure

The antimicrobial activity of the TO@NZ hybrid nanostructure was estimated by the minimum inhibitory concentration (MIC) against several bacteria. The MICs of TO@NZ were studied by the broth dilution assay in Brain Heart Infusion broth (BHI, Oxoid, UK) and the selected bacteria were two lactic acid bacteria, which commonly are used as starter cultures in cheese manufacturing, namely *Lactococcus lactis* sp. *lactis* ACA-DC127 and *Streptococcus thermophilus* ST112, and 3 pathogenic strains, namely *Staphylococcus aureus* ATCC1538, *Listeria monocytogenes* NCTC10527, and *Enterococcus faecalis* EF1. All the strains were obtained from the ACA-DC Culture Collection of Food Science and Human Nutrition Department of the Agricultural University of Athens. The strains were preserved on cryo-beads in cryovials of a cryopreservation system (TSC Ltd., Queensway Industrial Estate, 3–4 Arkwright Way, Scunthorpe DN16 1AL UK) at −80 °C, and overnight cultures were grown in BHI broth at 30 °C for *L. Lactis* ACA-DC127, and 37 °C for *S. thermophilus* ST112 and all pathogenic strains. For the broth dilution assay, five BHI broths with TO@NZ concentrations of 0.01, 0.025, 0.05, 0.075, and 0.1% (*w*/*v*) were made. The selected bacteria were inoculated into BHI broths, in a final concentration of approximately 10^6^ cfu/mL in the presence of the above different concentrations of TO@NZ hybrid nanostructure, and incubation was followed for 48 h at the appropriate temperature for each strain. Growth was assessed by measuring the absorbance at 625 mM and agar plating after incubation for 48 h at 30 °C for the *L. lactis* sp. *lactis* AC A-DC127 strain and at 37° for the other bacteria to determine viable cfu/mL. The MIC values were read as the lowest concentrations of TO@NZ at which bacterial growth was completely inhibited. In addition, to determine the minimal bactericidal concentration (MBC), the dilution representing the MIC and at least two of the more concentrated TO@NZ dilutions were plated and enumerated to determine viable cfu/mL. The MBC value was estimated as the lowest concentration that demonstrated a reduction (such as 99.9%) in cfu/mL of the strain tested.

#### 4.14.2. Antimicrobial Activity of Active Films Application on Cheese against *S. aureus*

The antimicrobial activity of active films on toxigenic strain *S. aureus* ATCC1538 growth in cottage cheese was studied considering the possible diffusion of essential oil from the film matrix into the soft cheese. The cottage cheese divided into 5 treatments consisting of (1) control uncoated sample (C); (2) sample coated with ALG/G film without the addition of the hybrid nanostructure active hybrid nanostructure TO@NZ; (3) sample coated with ALG/G/5TO@NZ film; (4) sample coated with ALG/G/10 TO@NZ films; and (5) sample coated with ALG/G/15TO@NZ. Cottage cheese for each treatment was weighed into 50±0.5 g portions and placed in a stomacher bag. The cheese was inoculated with a fresh suspension of pathogenic *S. aureus* ATCC1538 strain in a final count of 10^4^ cfu/g. The cheese was homogenized for 2 min by the stomacher (Seward 400; UK) and placed into sterile plastic petri-dish (9 cm diameter) as a thin layer. The film materials prepared as described above were applied to the upper surfaces of the cheese except for control (C); and the plates after being covered with their lid; were held at 10 °C for 10 days and sampled at days 0; 2; 5; 7; 10. The choice of time and temperature abuse of samples was selected as the most common conditions of unproperly preserved foodstuffs in the retail market; causing foodborne illness. At each sampling interval, duplicate plates from each treatment were aseptically opened and a 10 g-portion was weighed in a Stomacher-bag and homogenized in sterile peptone salt solution (Merck; Germany) in a stomacher (Seward 400; UK) for 1 min to make the initial dilution (10^−1^). Appropriate serial dilutions (10^−1^–10^−5^) were spread plated on Baird Parker agar with egg yolk tellurite emulsion (BPA; Merck; Germany) and incubated at 37 °C for 48 h for *Staphylococcus aureus* ATCC1538 counts.

### 4.15. Statistical Analysis

All the results listed in Table 1, Table 2, Table 3 and Table 4 are the mean values of measurements carried out to three samples for every kind of film. The statistical software SPSS ver. 20 was used for the interpretation of the experimental data. Mean values of all the measured properties were extracted based on the assumption of a confidence interval C.I. = 95% which is the most common value. Thus, the value of the statistical significance level was a = 0.05. Standard deviation values are also presented in such tables within parentheses beside the mean value. Finally, hypothesis tests were carried out to confirm that considering a different kind of film, every property has a statistically different mean value. Because of non-positive normality tests for all datasets, the non-parametric Kruskal–Wallis method was used. The results show that the mean values of all properties are statistically unequal.

## Figures and Tables

**Table 1:** Calculated values of Young’s (E) Modulus, ultimate tensile strength (σ_uts_) and % strain at break (ε_b_).

Code Name	E-Elastic Modulus (MPa)	σ_uts_ (MPa)	%ε
ALG/G	445.5 (63.8)	15.2 (2.4)	40.2 (4.7)
ALG/G/5NZ	755.6 (67.3)	22.7 (0.9)	24.7 (12.4)
ALG/G/10NZ	669.3 (24.3)	21.1 (5.9)	20.3 (2.7)
ALG/G/15NZ	785.3 (146.6)	23.1 (5.5)	23.1 (2.5)
ALG/G/5TO@NZ	739.4 (20.3)	20.9 (3.5)	28.4 (8.2)
ALG/G/10TO@ΝΖ	651.5 (76.2)	18.5 (2.9)	28.3 (6.6)
ALG/G/15TO@NZ	798.5 (177.5)	22.6 (1.4)	25.3 (2.5)

**Table 2:** Measured values of water-vapor transmission rate WVTR and oxygen transmission rate OTR. Calculated values of water diffusion coefficient D_WV_ and oxygen permeability coefficient Pe_O2_ for all obtained films.

Code Name	Film Thickness (mm)	WVTR (10^−6^) (gr∙cm^−2^∙s^−1^)	D_WV_ (10^−4^) (cm^2^∙s^−1^)	OTR (10^−4)^ (ml∙cm^−2^∙day^−1^)	Pe_O2_ (10^−7^) (cm^2^∙s^−1^)
ALG/G	0.11 (0.01)	2.45 (0.14)	5.96 (0.45)	111,939 (234)	15.70 (0.21)
ALG/G/5NZ	0.07 (0.01)	3.00 (0.45)	5.40 (0.11)	70,476 (124)	5.71 (0.10)
ALG/G/10NZ	0.08 (0.01)	2.31 (0.21)	3.97 (0.42)	85,456 (174)	9.23 (0.19)
ALG/G/15NZ	0.09 (0.01)	2.47 (0.25)	5.55 (0.67)	127,556 (435)	15.3 (0.52)
ALG/G/5TO@NZ	0.08 (0.01)	2.36 (0.21)	4.06 (0.11)	93,984 (205)	9.06 (0.20)
ALG/G/10TO@NZ	0.10 (0.01)	2.26 (0.16)	5.16 (0.23)	90,549 (345)	6.99 (0.27)
ALG/G/15TO@NZ	0.13 (0.01)	2.28 (0.27)	6.89 (0.74)	78,476 (234)	11.8 (0.35)

**Table 3:** Antioxidant activity of all obtained films.

Code Name	% Antioxidant Activity 24 h
ALG/G	8.7 (0.6)
ALG/G/5NZ	7.7 (2.7)
ALG/G/10NZ	10.4 (1.9)
ALG/G/15NZ	15.1 (2.0)
ALG/G/5TO@NZ	17.3 (1.3)
ALG/G/10TO@NZ	25.3 (3.9)
ALG/G/15TO@NZ	46.4 (4.3)

**Table 4:** Minimum inhibitory concentrations (MIC) and minimum bactericidal concentrations (MBC) of TO@NZ hybrid nanostructure against lactic acid bacteria and pathogens (% *w*/*v*).

Bacteria (10^6^ cfu/mL)	MIC	MBC
*Lactococcus lactis* ssp. *lacts* ACA-DC127	0.025 (0.1)	0.025 (0.1)
*S. thermophilus* ACA-DC112	0.025 (0.1)	0.025 (0.1)
*S. aureus* ATCC1538	0.05 (0.4)	0.05 (0.4)
*L. monocytogenes* NCTC10527	0.1 (0.2)	0.1 (0.2)
*E. faecalis* EF1	0.1 (0.3)	0.1 (0.4)

**Table 5:** Code names and amounts of ALG, G, NZ and TO@NZ used for the preparation of films.

Code Name	ALG (g)	G (g)	NZ (g)	TO@NZ (g)
ALG/G	2	1	-	-
ALG/G/5NZ	2	1	0.15	-
ALG/G/10NZ	2	1	0.30	-
ALG/G/15NZ	2	1	0.45	-
ALG/G/5TO@NZ	2	1	-	0.15
ALG/G/10TO@NZ	2	1	-	0.30
ALG/G/15TO@NZ	2	1	-	0.45

## Data Availability

The datasets generated for this study are available on request to the corresponding author.

## Supplementary Materials

The following supporting information can be downloaded at: https://www.mdpi.com/article/10.3390/gels8090539/s1, Table S1. LC-MS/MS analysis of thyme oil as received used for the modification of NZ based hybrids; Table S2. LC-MS/MS analysis of remaining thyme oil after the first stage distillation process for the modification of NZ based hybrids.
