# Supplementary material for: The Increase of Soft Cheese Shelf-Life Packaged with Edible Films Based on Novel Hybrid Nanostructures
Source: Gels. 2022 Aug 26;8(9):539. doi: 10.3390/gels8090539 (PMC9498775; doi:10.3390/gels8090539)
Supplement: Supplementary file 1 [file gels-08-00539-s001.zip › gels-1866763-supplementary.pdf]

Table S1. LC-MS/MS analysis of thyme oil as received used for the modification of NZ based hybrids

Peak	Label	Area Sum %
1	Cpd 1: .ALPHA.-PINENE, (-)-; C10 H16; 6.658	0.81
2	Cpd 2: Camphene; C10 H16; 7.148	0.25
3	Cpd 3: Sabinene; C10 H16; 8.171	0.11
4	Cpd 4: .beta.-Myrcene; C10 H16; 8.864	0.56
5	Cpd 5: Cyclohexene, 1-methyl-4-(1-methylethenyl)-, (R)-; C10 H16; 9.298	0.09
6	Cpd 6: .ALPHA. TERPINENE; C10 H16; 9.802	0.72
7	Cpd 7: Cyclohexane, 1-methyl-3-(1-methylethenyl)-, cis-; C10 H18; 9.998	0.12
**8**	**Cpd 8: Benzene, 1-methyl-4-(1-methylethyl)-; C10 H14; 10.145 ή p-cymene**	**12.28**
**9**	**Cpd 9: D-Limonene; C10 H16; 10.341**	**16.54**
10	Cpd 10: .gamma.-Terpinene; C10 H16; 11.616	0.34
11	Cpd 11: .alpha.-terpinolene; C10 H16; 12.890	0.23
12	Cpd 12: Linalool; C10 H18 O; 13.497	1.67
13	Cpd 13: Camphor; C10 H16 O; 15.292	0.11
14	Cpd 14: Cyclohexanol, 1-methyl-4-(1-methylethenyl)-; C10 H18 O; 15.390	0.08
15	Cpd 15: Bicyclo[2.2.1]heptan-2-ol, 1,7,7-trimethyl-, exo-; C10 H18 O; 15.845	0.1
16	Cpd 16: endo-Borneol; C10 H18 O; 16.265	0.41
17	Cpd 17: .ALPHA.-TERPINEOL; C10 H18 O; 17.462	4.21
18	Cpd 18: Cyclohexanol, 1-methyl-4-(1-methylethylidene)-; C10 H18 O; 17.778	0.52
19	Cpd 19: Fenchyl acetate; C12 H20 O2; 18.821	0.26
20	Cpd 20: Cyclohexanol, 2-methylene-3-(1-methylethyl)-, acetate, cis-; C12 H20 O2; 20.228	0.12
21	Cpd 21: Bicyclo[2.2.1]heptan-2-ol, 1,7,7-trimethyl-, acetate, (1S-endo)-; C12 H20 O2; 21.831	2.89
**22**	**Cpd 22: Phenol, 5-methyl-2-(1-methylethyl)-; C10 H14 O; 22.273 THYMOL**	**32.54**
**23**	**Cpd 23: Phenol, 5-methyl-2-(1-methylethyl)-; C10 H14 O; 22.651**	**24.16**
24	Cpd 24: Tetradecane; C14 H30; 26.754	0.11
25	Cpd 25: TRANS(.BETA.)-CARYOPHYLLENE; C15 H24; 27.251	0.36
26	Cpd 26: Hexadecane; C16 H34; 34.638	0.17
27	Cpd 27: Octadecane; C18 H38; 41.781	0.07
28	Cpd 28: Hexadecanoic acid, 2-hydroxy-1-(hydroxymethyl)ethyl ester; C19 H38 O4; 60.105	0.19

Table S2. LC-MS/MS analysis of remaining thyme oil after the first stage distillation process for the modification of NZ based hybrids

Peak	Label	Area Sum %
1	Cpd 1: Camphene; C10 H16; 7.142	0.18
2	Cpd 2: Benzene, 1-methyl-4-(1-methylethyl)-; C10 H14; 10.131	0.99
3	Cpd 3: D-Limonene; C10 H16; 10.292	0.97
4	Cpd 4: Linalool; C10 H18 O; 13.492	0.69
5	Cpd 5: 3-Cyclohexen-1-ol, 1-methyl-4-(1-methylethyl)-; C10 H18 O; 14.942	0.21
6	Cpd 6: Camphor; C10 H16 O; 15.278	0.15
7	Cpd 7: Cyclohexanol, 1-methyl-4-(1-methylethenyl)-; C10 H18 O; 15.383	0.17
**8**	Cpd 8: 15.838	0.19
**9**	Cpd 9: Bicyclo[2.2.1]heptan-2-ol, 1,7,7-trimethyl-, exo-; C10 H18 O; 16.258	0.54
10	Cpd 10: 3-Cyclohexen-1-ol, 4-methyl-1-(1-methylethyl)-; C10 H18 O; 16.825	0.11
11	Cpd 11: .ALPHA.-TERPINEOL; C10 H18 O; 17.463	1.65
12	Cpd 12: Cyclohexanol, 1-methyl-4-(1-methylethylidene)-; C10 H18 O; 17.778	0.54
13	Cpd 13: 18.611	0.09
14	Cpd 14: Fenchyl acetate; C12 H20 O2; 18.821	0.54
15	Cpd 15: Ascaridole; C10 H16 O2; 20.249	0.19
16	Cpd 16: Isobornyl acetate; C12 H20 O2; 21.748	0.36
17	Cpd 17: Bicyclo[2.2.1]heptan-2-ol, 1,7,7-trimethyl-, acetate, (1S-endo)-; C12 H20 O2; 21.867	3.38
18	**Cpd 18: Phenol, 5-methyl-2-(1-methylethyl)-; C10 H14 O; 22.294**	**46.61**
19	**Cpd 19: Phenol, 5-methyl-2-(1-methylethyl)-; C10 H14 O; 22.679**	**40.09**
20	Cpd 20: 25.592	0.18
21	Cpd 21: Tetradecane; C14 H30; 26.747	0.15
**22**	Cpd 22: TRANS(.BETA.)-CARYOPHYLLENE; C15 H24; 27.251	0.33
**23**	Cpd 23: 29.282	0.18
24	Cpd 24: CARYOPHYLLENE OXIDE; C15 H24 O; 33.672	0.1
25	Cpd 25: Hexadecane; C16 H34; 34.639	0.21
26	Cpd 26: 48.132	0.37
27	Cpd 27: 48.426	0.39
28	Cpd 28: 60.105	0.43
